# Protective effects of stem bark of *Harungana madgascariensis* on the red blood cell membrane

**DOI:** 10.1186/1472-6882-13-98

**Published:** 2013-05-10

**Authors:** Prosper Cabral Nya Biapa, Horea Matei, Ştefana Bâlici, Julius Eyong Oben, Jeanne Yonkeu Ngogang

**Affiliations:** 1 Department of Biochemistry, Faculty of Sciences, University of Dschang, Dschang, Cameroon; 2 Department of Cell and Molecular Biology, “Iuliu Haţieganu” University of Medicine and Pharmacy, Cluj-Napoca, Romania; 3 Department of Biochemistry, Faculty of Sciences, University of Yaoundé I, Yaoundé I, Cameroon; 4 Faculty of Medicine and Biomedical Sciences, University of Yaounde I, Yaounde I, Cameroon

**Keywords:** Anemia, Water permeability, NMR, *Harungana madagascariensis*

## Abstract

**Background:**

Anemia is a condition that has multiple origins. One such origin is the destruction of red blood cells’ (RBCs) membrane induced by free radicals. Treatment of anemia could therefore be enhanced by the use of free radicals’ scavengers potentially found in some medicinal plants. In this study, the protective effect of *Harungana madagascariensis* on the RBCs’ membrane physiology was investigated *in vitro* and *in vivo*.

**Methods:**

*In vitro* hemolytic anemia was induced by incubation of fresh human RBCs with carbontetrachloride (CCl_4_) in Olive oil (Oo). Relaxation times of protons excited at 20 MHz (Carr-Purcell-Meiboom-Gill pulse sequence) in the absence or presence of paramagnetic Mn^2+^ ions (*T*_*2i*_ for “extracellular” water and *T*_*2a*_ for “intracellular” water, respectively) were determined at several temperatures (25–37°C) via Nuclear Magnetic Resonance (NMR) on a Bruker Minispec spectrometer. Water exchange times (T_e_) were consequently calculated using the Conlon-Outhred equation: 1/T_e_ = (1/T_2a_) – (1/T_2i_). Morphological characteristics (mean cell volume, V, and cell surface area, A) were determined by photonic microscopy and the RBCs’ diffusional water permeability (*P*_*d*_) was calculated as P_d_ = (1/T_e_)*(V_a_/A), where V_a_ is the aqueous volume in the RBC and is about 0.7 of the cell volume (V). The activation energy of the diffusional process (E_a_) for the respective temperature range was estimated using the Arrhenius modified equation k = A(T/T_0_)^n^*e^-Ea/RT^. Inhibition of the water diffusion induced by incubation *with para*-chloro-mercuribenzoic acid (PCMB) at 25, 30 and 37°C was calculated as I(%) = [(Pd control – Pd sample)/Pd control]*100.

To investigate the protective influence of the extract on the RBC membrane, inhibition of the water permeability was evaluated on membranes pre-incubated with the *Harungana madagascariensis* extract. Male rats were used in *in vivo* investigations. Malondialdehyde (MDA) and cholesterol in the RBC membrane were estimated by induction of lipid peroxidation while the antioxidant properties of catalase (CAT) and superoxide dismutase (SOD) on the membrane were evaluated in regard to their antioxidant properties on the membrane.

**Results:**

*T*_*2a*_ significantly decreased at each temperature. *Te* results were higher in both RBCs and RBCs + extract groups incubated with PCMB compared to non-incubated controls, but differences were not statistically significant. A high percentage (73.81 ± 7.22) of RBCs pre-incubated with the extract presented the regular biconcave shape. Inhibition by PCMB of the RBCs’ membrane water permeability was increased at 30°C and decreased in the presence of extract (25°C and 37°C), while *Ea* decreased from 30.52 ± 1.3 KJ/mol to 25.49 ± 1.84 KJ/mol. Presence of the *Harungana madagascariensis* extract normalized the SOD and CAT activities as well as the MDA and membrane cholesterol concentrations altered by the CCl_4_-induced oxidative stress.

**Conclusion:**

*Harungana madagascariensis* could protect the RBCs’ membrane through its antioxidative properties.

## Background

The erythrocyte membranes refer to the lipid bilayer (phospholipids and cholesterol) containing the transmembrane proteins of the cytoskeleton and an assembly of proteins regularly interconnected [1,2]. Multiple proteins make up the erythrocyte membrane cytoskeleton, which interacts with both the lipid bilayer and transmembrane proteins to give the red blood cell its characteristics strength and integrity of major intrinsic proteins. Ankyrin provides the primary linkage between α- and β-spectrin tetramers and the cytoplasmic domain of the major integral protein band 3, a relationship enhanced by interactions with protein 4.2. The junctional complexes formed by spectrin tetramers and their interaction with tropomyosin, adducin, actin and other proteins ensure the stability between the cytoskeleton and the lipid bilayer [2]. In some abnormal conditions of the RBCs membrane, such as hereditary spherocytosis (HS), hereditary elliptocytosis (HE), hereditary pyropoikilocytosis (HPP) or the Southeast Asian Ovalocytosis (SAO), these membrane proteins are usually defective because of their specific inherited gene mutations [3]. The high permeability of RBC membranes is due to the presence of water channel proteins called aquaporins (AQP) [4]. An important characteristic of RBCs’ water diffusional permeability is its inhibition by the PCMB [5]. There are two basic strategies for measuring water exchange through the RBC membranes: the non-stationary method via volume changes and the stationary method using NMR techniques [6].

Self*-*administered drugs, auto-immunity, immunoallergy, parasitic and viral infections could also lead to oxidative stress and defective RBCs [7]. Oxidative stress results from an imbalance between the formation and neutralization of pro-oxidants [8,9]. It is well known that carbontetrachloride as well as phenylhydrazine induces RBC damages [10,11]. Cells have therefore developed antioxidant mechanisms to quench the free radicals but when the generation of free radicals exceeds the scavenging capacity of the cell, the excessive free radicals seek stability through electron sharing with biological macromolecules such as proteins, lipids and DNA. This is why in healthy human cells this process results in the induction of lipid peroxidation, which in turn leads to atherosclerosis, cardiovascular diseases, ageing and inflammatory diseases [12]. Free radicals are known to be scavenged by synthetic antioxidants, but this procedure can sometimes generate adverse side effects leading to carcinogenicity. Search for effective natural antioxidants has become crucial [13].

World Health Organization (WHO) encourages the use of medicinal plants in the treatment of diseases [14]. *Harungana madagascariensis* is a medicinal plant traditionally used to fight anemia in Cameroon. Previous works have shown the anti-malarial activity of its methanolic extract [15]. Harunganin, harongin anthrone and 1, 7-dihydroxyxanthone were isolated from the stem bark of this plant and their structures were elucidated by spectroscopic analysis [16]. Preceding works conducted in our laboratory included the phytochemical screening and *in vitro* antioxidant properties of the hydro-ethanolic bark extract as well as the *in vivo* anti-anemic activity of the same extract after the induction of hemolytic anemia in rats using phenylhydrazine (PHZ) [17,18]. In this study, we will further investigate the protective properties of the hydro-ethanolic bark extract of *Harungana madagascariensis* on the RBCs’ membrane physiology.

## Methods

### Animals

The scientific committee of the University of Yaoundé I and the Cameroon National Ethics Committee approved the experimental procedures. Male rats (200 g) maintained on a 12h light/dark cycle at room temperature (26°C) with a relative humidity of 25% were allowed free access to water and food (made-up with maize, fish, salt, vitamins, soybean and oil).

### Plant material

The stem bark of *Harungana madagascariensis*, from the family of Hypericaceae, has been collected in Okola-Yaounde, in January 2010. The plant has been authenticated at the National Herbarium of Cameroon in Yaounde (Voucher specimen N° 4224 HNC).

### Extraction

After being washed and dried at room temperature, the collected plant was powdered and sieved. 100 g were soaked for about 48 hours in 2 litres of water - ethanol mixture (1:1 ratio) and then the suspension was filtered. Remaining residues were re-extracted as shown above. The total filtrate was concentrated using a rotary evaporator (Heidolph WB 2000). Water was further evaporated in an oven at 50°C, giving 45% as yield of extraction. This procedure was chosen according to previous works with this extract [17,18].

### Blood sample collection

A fresh blood sample of a healthy 34 years old Cameroonian was used after informed consent from the subject was obtained. The procedure was approved by the ethics committee of the University of Medicine and Pharmacy in Cluj-Napoca, Romania. 20 mL of blood collected on Ethylenediaminetetraacetic acid (EDTA) at 4°C in the Neuro-Pediatric Clinic Center in Cluj-Napoca, Romania were used for the *in vitro* experiments.

### *In vitro* experiments

#### Purification of red blood cells

The method described by Benga et al. was used [19]. Briefly, from the blood sample refrigerated immediately after collection, RBCs have been isolated by three consecutive centrifugations followed by washings in medium S (150 mM.L^-1^ NaCl, 5.5 mM.L^-1^ glucose, 5 mM*L^-1^ HEPES, pH 7.4).

### Morphological measurements of RBCs

#### Experiment

Washed RBCs membranes’ damage was induced using CCl_4_ in Oo. Many groups have been formed against the washed RBCs control (Table 1A). The 100 mM PCMB solution was made up by mixing 0.035716 g PCMB with 300 μL of 1 M NaOH. After homogenization wash buffer (WB) was added up to 1 mL. The photosensible solution was kept at 4°C covered in aluminium sheet until use. Samples containing 600 μL RBCs pre-incubated with extract or CCl_4_, 60 μL PCMB 100 mM and 5.34 mL WB (final PCMB concentration 1 mM) were incubated for 1 h at 37°C under continuous stirring. After incubation, each sample was washed 3 times.

**Table 1:** **Experimental groups for the morphological measurements of RBCs, the RBCs water permeability and the *in vivo *study of the extract against the oxidative stress**

**Groups**	**Experiment**
**A- Experimental groups for the morphological measurements of RBCs**	
1	RBCs (600 μL) + Extract in Oo 1:1 (21 μL) + WB,Prein 37°C- 30 min, In 37°C- 1 h
2	RBCs (600 μL) + CCl_4_ (6 μL) (in Oo 1:1) + WB, In 37°C- 1 h
3	RBCs (600 μL) + CCl_4_ (6 μL) + WB, In 37°C- 1 h
4	RBCs (600 μL) + Extract in Oo 1:1 (21 μL) + WB, Prein 37°C- 30 min, CCl_4_ (6 μL) (in Oo 1:1), In 37°C - 1 h
5	RBCs (600 μL) + WB + PCMB 1 M (60 μL), In 37°C - 1 h
6	RBCs (600 μL) + Extract in Oo 1:1 (21 μL) + WB, Prein 37°C - 30 min, PCMB (60 μL) (in Oo 1:1), In 37°C - 1 h
**B- Experimental groups for evaluating the RBCs water permeability in the presence or no of PCMB: NMR measurements**	
1	RBCs (1200 μL) + WB, Prein 37°C - 30 min, PCMB 1 M (60 μL), In 37°C - 1 h
2	RBCs (1200 μL) + extract in Oo, 1:1 (42 μL) + WB, In 37°C - 1 h
**C- Experimental groups for the *in vivo *study of the extract effects in relation with the lipid peroxidation and the oxidative stress on the RBCs**	
1	Oo each day for 1 week (1 mL/200 g bw/day)
2	Oo each day for 1 week (1 mL/200 g bw/day), CCl_4_ (2 mL/kg bw/day) in Oo on day 7
3	extract (0.016 g/kg bw/day) 1 week and CCL_4_ (2 mL/kg bw/day) in the Oo on day 7
4	extract (0.032 g/kg bw/day) 1 week and CCl_4_ (2 mL/kg bw/day) in the Oo on day 7
5	extract (0.064 g/kg bw /day) 1 week and CCl_4_ (2 mL/ kg bw/day) in the Oo on day 7

WB = washed buffer, Prein = pre-incubation, In = incubation, Oo: Olive oil, The final volume of 6 mL in each tube was completed with wash buffer. The extract volume (0.064 g of extract / kg bw) prepared with Oo (1:1, m/v - according to previous work) was calculated for a final CCl_4_ or PCMB tube concentration of 1 mM.

#### Measurements of RBCs

For the estimation of RBC cells’ number per mm^3^ (N), 16 μL of RBC: bovine serum albumin (BSA) 0.5% (1:1) were diluted in 10 ml WB and gently homogenized. A drop of the solution was placed on the Thoma slide and covered with thin glass coverslip so that refraction rings became apparent. The slide was examined under a Nikon Eclipse 80i microscope with a color video camera and the number of RBC cells (N) entirely placed within 16 large (256 small) grid squares were counted. For determining the hematocrit (Ht %) the RBC:BSA 0,5% (1:1) suspension was drawn into three capillaries and sealed, then centrifuged 10 minutes on a Hawksley microcentrifuge. The sedimented hematocrit (%) was measured on the Hawksley scale and expressed as mean value of the three capillary samples. For diameter (D) measurements 5 μL of the RBC:BSA 0,5% (1:1) suspension were dropped in a thin BSA 0.5% layer on a glass slide and homogenized, then the area was delimited with a thin nylon thread, sealed with a glass coverslip and examined under the Nikon Eclipse 80i microscope. Cell volume (V) in mm^3^ was estimated as V = Ht*10/N. The cell surface was derived from the formula: S= 4V/D + ЛD^2^/2.

### RBCs water permeability

The method for the RBCs water permeability assessment has been previously described [6,20].

#### Washed RBCs treatments and NMR measurements

Washed RBCs were treated in the presence and absence of the PCMB inhibitor as presented in Table 1B. The fresh PCMB solution was prepared as above and RBCs were washed 3 times with WB and then centrifuged. The incubated washed RBCs (Table 1B) were split prior to being subjected to NMR. For the first part, used to determine longitudinal relaxation times of protons excited at 20 MHz (Carr-Purcell-Meiboom-Gill pulse sequence) on a Bruker Minispec spectrometer [6,20] in the presence of paramagnetic Mn^2+^ ions (*T*_*2a*_ for “extracellular” water), the NMR tube was formed by gently mixing 200 μL of washed RBCs with 200 μL BSA 0.5% in WB and 200 μL of doping solution containing 40 mM MnCl_2_ and 100 mM NaCl. The second part was further centrifuged for 1 h at 50,000 g and was used (washed RBCs only) to determine ^1^H^+^ transversal relaxation time (*T*_*2i*_ for “intracellular” water). The thermostat was progressively set for temperatures of 25°C, 30°C and 37°C and *T*_*2i*_ and *T*_*2a*_ measurements recorded. Water exchange times (T_e_) were consequently calculated using the Conlon-Outhred equation: 1/T_e_ = (1/T_2a_) – (1/T_2i_). Based on the exchange times and morfological determinants, RBCs’ diffusional water permeability (P_d_) was calculated as P_d_ = (1/T_e_)*(V_a_/A). The activation energy of the diffusional process (E_a_) for the respective temperature range was estimated from experimental data using the Arrhenius modified equation k = A(T/T_0_)^n^*e^-Ea/RT^ that makes explicit the temperature dependence of the pre-exponential factor [5]. Inhibition of the water diffusion induced by incubation with PCMB at 25, 30 and 37°C was calculated as I (%) = [(Pd control – Pd sample)/Pd control] × 100.

All NMR tests followed the same procedure, including the non-incubated washed RBCs used as controls.

### *In vivo* experiments on rats

Table 1C illustrates different groups formed for this investigation. Olive oil was used as carrier for the *H. madagascariensis* hydroethanolic extract. 2 mL/kg body weight (bw) of CCl_4_ in Oo (1:1) were given orally on the seventh day to induce lipid peroxidation. RBCs membrane’s lipid extraction was conducted using the method described by Folch et al [21]. Briefly, the tissue (precipitate) was homogenized with chloroform/methanol (2/1) to a final volume 20 times the volume of the tissue sample. After dispersion, the whole mixture was stirred 15–20 min in an orbital shaker at room temperature. The homogenate was centrifuged at 3000 rpm to recover the liquid phase. This process was repeated 2 times. The solvent was washed with 1% NaCl (1:1) solution. After stirring, the mixture was centrifuged at 2000 rpm to separate the two phases. The upper phase was eliminated by siphoning and the methanol/water (1/1) mixture was added to the lower phase and centrifugated at 3000 rpm. The lower chloroform phase containing the lipid fraction was evaporated under vacuum in a rotary evaporator.

The extract’s effect against lipid peroxidation was quantified by the MDA and membrane cholesterol levels [22,23]. 400 μL of thiobarbituric acid (TBA) reagent (20% trichloroacetic acid, 0.375% TBA, 0.01% BHT (Butyl-Hydroxy-Toluene) and 1N HCl) was added to 100 μL of serum. The mixture was incubated 15 min in a boiling water bath. After cooling, the suspension was centrifuged at 3000 rpm for 10 minutes. The supernatant was then separated and absorbance was measured at 532 nm. The MDA concentration was determined by the specific absorbance coefficient (1.34×10^5^ mol/cm^3^).

The lipid extract obtained using Folch’s method was diluted (1:10) with acetone-ethanol (1:1) mixture and centrifuged at 3400 rpm for 20 min. 0.4 mL of the mixture was diluted with 6 mL FeSO_4_ . 2 ml of concentrated sulfuric acid were added and mixed thoroughly. After 10 min at rest, the absorbance of the solutions of the tube was read at 490 nm. The standard cholesterol solution was treated in the same manner and all readings of the absorbance were performed against a blank consisting of 0.4 ml acetone-ethanol mixture. The RBC membrane cholesterol was estimated as follows:

$$\text{RBCMembraneCholesterol}\left(\frac{\mathrm{mg}}{\mathrm{\mu l}}\right)=\frac{\text{DOtest}}{\text{DOstd}}\times \text{StdConc}\times \text{DilutionFactor}$$

The antioxidant activity of the extract on the RBCs membrane was also estimated by measuring CAT and SOD activities [24,25]. The measurement of total SOD activity was performed according to Misra and Fridovich method [25], based on the inhibition of epinephrine autoxidation. 0.2 mL distilled water and 2.5 mL sodium carbonate buffer 0.05 M, pH 10.2.were added to the 0.3 ml buffered epinephrine to initiate the reaction. The absorbance at 480 nm was read for 150 s at 30 s intervals against a blank made up of 2.5 mL buffer, 0.3mL epinephrine and 0.2 mL distilled water. The following equation allowed the calculation of the SOD activity

$$\text{SOD}\left(\frac{\text{unit}}{\mathrm{mg}}\text{proteins}\right)=\frac{\text{SOD}\left(\text{unit}/\mathrm{mL}\right)}{\mathrm{Pr}\text{oteins}\left(\mathrm{mg}/\mathrm{mL}\right)}\times \text{DilutionFactor}$$

The evaluation of CAT activity was performed by mixing 2.5 mL H_2_O_2_ 30 mM with 0.5 mL hemolysate. The disappearance of peroxide with the absorbance decrease in 30 s steps for 90 s was followed spectrophotometrically at 240 nm against a blank made up with 3 mL H_2_O_2_. One unit decomposes one micromole of H_2_O_2_ per minute at 25°C and pH 7.0 under the specified conditions.

$$\mathrm{CAT}\left(\frac{\text{unit}}{\mathrm{mg}}\text{proteins}\right)=\frac{\mathrm{\Delta A}/\min\times 30000\mathrm{units}}{40\mathrm{cm}/M\times \mathrm{mg}\mathrm{Pr}\text{oteins}}\times \text{DilutionFactor}$$

## Results

**Figure 1:**
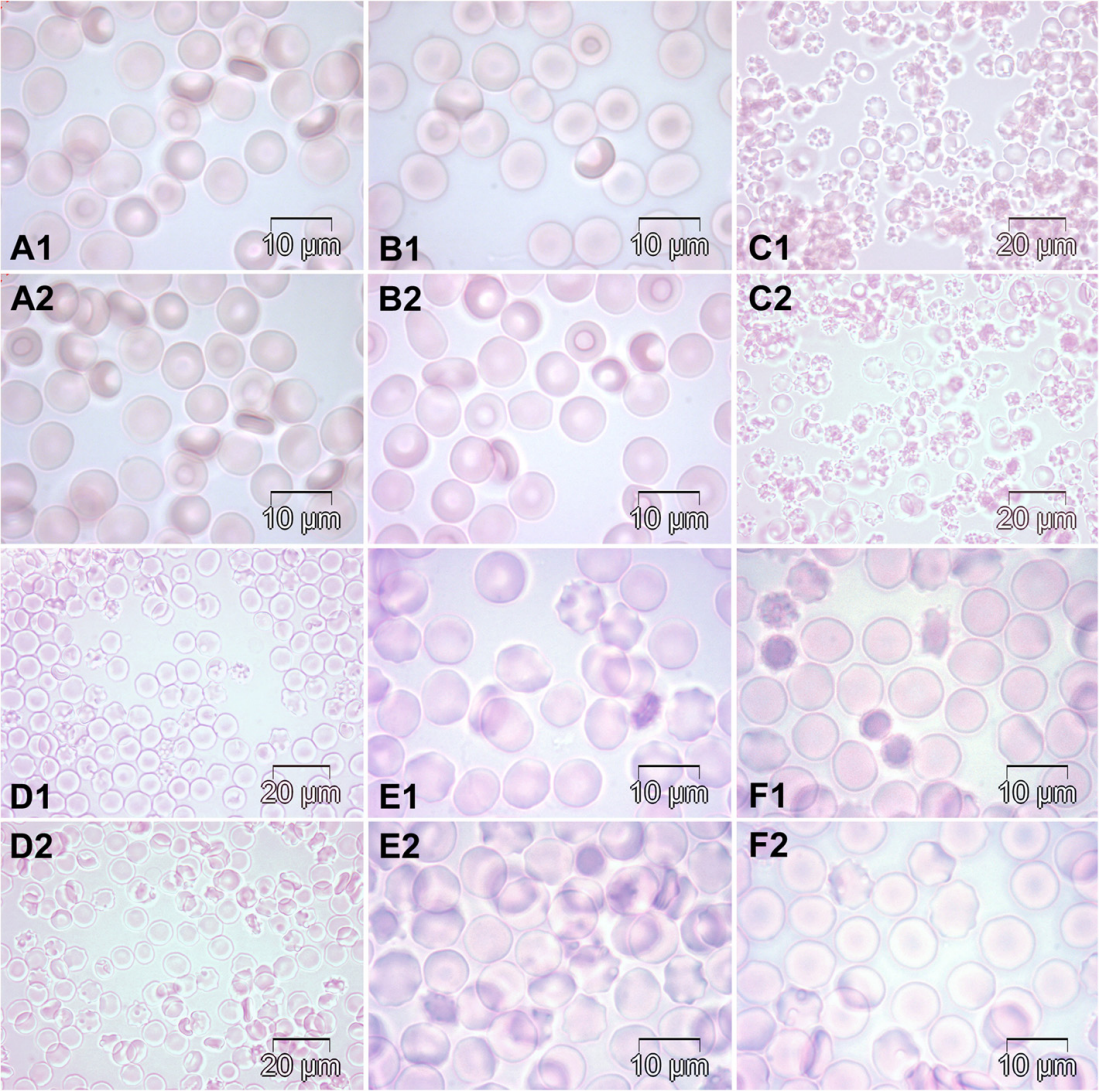
**Bright field microscopy images presenting morphological changes of RBCs.** Note the normal RBCs’ morphology in the control group (panels **A1-2**, 100×) and the degrees of morphological alteration seen in different experimental groups: RBCs pre-incubated with extract and incubated with Oo (panels **B1-2**, 100×); RBCs incubated with CCl4 (panels **C1-2**, 40×); RBCs incubated with CCl4+ Oo (panels **D1-2**, 40×); RBCs incubated with PCMB (panels **E1-2**, 100×) and RBCs pre-incubated with extract followed by incubation with PCMB (panels **F1-2**, 100×).

Figure 1B to 1F show pictures of RBCs in different conditions against Figure 1A representing the non-incubated controls. It appears that the biconcave shape which characterizes the healthy RBCs is seen in Figure 1A controls as well as in Figure 1B, where RBCs were pre-incubated with the extract at 37°C. In contrast, Figure 1C presented damaged RBCs when incubated with CCl_4_. Moreover, red color (hemolytic signs) has been physically observed in tubes containing the washed RBCs incubated with CCl_4_. Hemolysis was not that evident when Oo was added to CCl_4_ before incubation (Figure 1D). It is apparent from Figure 1E and 1F that RBCs were less damaged when previously incubated with the extract before adding the PCMB. Nevertheless, some abnormalities appeared on several RBCs membranes.

Evaluated morphological parameters of the control RBCs were: V_a_/A = 0.46 ± 0.11 μm, Ht(%) = 45.5 ± 0.0%, N = 5.12 ± 0.35 million/mm^3^, A = 135 ± 4.8 μm^2^ and V = 88.9 ± 0.0 μm^3^. The percentages of healthy RBCs under multiple conditions are shown in Table 2. It appears from Table 2 that when RBCs were incubated with extract in Oo, 100% of these cells remained normal, while when incubated with CCl_4_ + Oo or CCl_4_ exclusively, the RBCs became less healthy. The same observation was made when the RBCs were incubated either with PCMB or extract Oo + PCMB respectively.

**Table 2:** Percentage of Healthy Red Blood Cells under different groups

**Groups**	**Percentage of healthy RBCs**
RBCs + Extract + Oo + PCMB	73.81 ± 7.22 ^a^
RBCs + PCMB	61.75 ± 8.66 ^b^
RBCs + CCl_4_Oo	9.76 ± 0.00 ^c^
RBCs + Extract Oo + CCl_4_Oo	27.28 ± 053 ^d^
RBCs + CCl_4_	5.43 ± 0.00 ^e^
RBCs + Extract + Oo	100.00 ± 000 ^f^

Oo = Olive oil; PCMB = Para – Chloromercuribenzoic acid; CCl_4_ = Carbon Tetrachloride; NP = total number of picture; One-way ANOVA followed by LSD. Values with different letters affected means significantly different at P<0.01.

**Figure 2:**
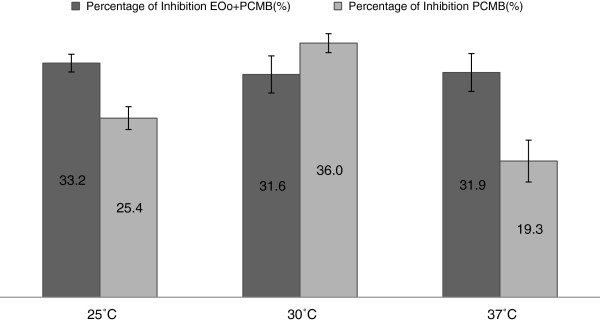
**Percentage of inhibition for the water diffusion permeability induced by PCMB.** EOo = extract +Oo, PCMB = Para-chloromercuribenzoic acid, washed RBCs were used in each group.

Table 3 and Figure 2 present the assessment of water diffusional exchange times (*Te*) and water diffusional permeability (*Pd),* as well as the degree of inhibition induced by PCMB. When compared to the standard *Pd* (G), no noticeable difference was observed between the control *Pd* (B), Pd (EOo + PCMB) (D) and *Pd* (PCMB) (F) at the same temperature. A similar observation was made when comparing control *Te* and *Te* (EOo + PCMB) (C) or *Te* (PCMB) (E).

**Table 3:** **Water diffusion exchange times (*Te) *and permeability rate (*Pd) *through the RBC membranes at 25, 30 and 37°C**

**Goups Temp**	**A**	**B**	**C**	**D**	**E**	**F**	**G**
25°C	11.1 ± 0.12	4.2 ± 0.15	16.6 ± 0.74	2.8 ± 0.66	14.8 ± 0.88	3.1 ± 0.74	4.1 ± 0.04
30°C	9.3 ± 0.16	4.9 ± 0.25	13.6 ± 1.02	3.4 ± 1.04	14.6 ± 0.6	3.2 ± 1.18	4.8 ± 0.04
37°C	7.6 ± 0.16	6.1 ± 0.25	11.1 ± 1.02	4.9 ± 1.04	9.4 ± 0.6	4.2 ± 1.18	6.0 ± 0.04

*Paired Student’s t test*.

A = *Te* control (ms), B = Pd control (x10^3^cm/s), C = *Te* (EOo+PCMB) (ms),

D = Pd (EOo+PCMB) (x10^3^cm/s), E = *Te* (PCMB) (ms), F = Pd (PCMB) (x10^3^cm/s),

G = Pd standard (x10^3^cm/s); EOo = extract +Oo, PCMB = Para-chloromercuribenzoic acid,

*Te* = water diffusion exchange time; *Pd* = Diffusional permeability; *Temp*: temperature.

Also *Pd* (EOo + PCMB) (D) at 30°C and 37°C is closer to control *Pd* and standard *Pd* values than *Pd* (PCMB) (F). At 30°C the percentage of inhibition for the water diffusional permeability of the PCMB-incubated RBCs was higher as compared to that of the extract-preincubated PCMB-incubated RBCs, while at 25°C and 37°C water diffusional permeability was higher for the extract-preincubated group not treated with PCMB. In addition, the *Ea* varied noticeably from 30.52 ± 1.3 KJ/mol to 25.49 ± 1.84 KJ/mol respectively.

The anti-peroxidative and antioxidant properties of the extract through the RBCs membrane are illustrated in Table 4. No significant difference was observed between controls’ MDA concentration (0.005 μmol/g) and that of the extract dose-dependent groups with values varying between 0.005 μmol/g and 0.0052 μmol/g, but significant difference was recorded when compared to the group that was treated with CCl_4_ + Oo.

**Table 4:** Variation of RBCs’ membrane cholesterol concentration, malondialdehyde, catalase and superoxide at different extracts’ concentration

**Groups**	**Oo**	**CCl4+Oo**	**0.016 g/kg**	**0.032 g/kg**	**0.064 g/kg**
Catalase (U/L)	47.90 ± 1.20	6.14 ± 0.5*	49.60 ± 1.80	51.10 ± 3.60	48.40 ± 1.80
Superoxide dismutase (U/L)	419.94 ± 6.20	169.33 ± 7.50*	415.80 ± 6.80	430.30 ± 9.60	426.32 ± 5.80
Membrane cholesterol (mg/μL)	5 ± 0.20	11 ± 1.50*	7.5 ± 0.80	7.51 ± 0.60	7.49 ± 0.80
Malondialdehyde(μmol /g protein)	0.005 ± 0.0	0.01 ± 0.0*	0.005 ± 0.0	0.0055 ± 0.0	0.0052 ± 0.0

* significantly different at *P<0.05*. One-way ANOVA followed by LSD, Oo: Olive oil.

Previous extract administration to rats was shown to stabilize the activities of CAT and SOD compared to controls that received Oo (Table 4).

## Discussion

Free radicals are critically involved in various pathological conditions such as cancer, cardiovascular disorders, arthritis, inflammation and liver diseases [26]. Under normal physiological conditions, low concentrations of lipid peroxidation products are found in tissues and cells. In the presence of oxidative stress, more lipid peroxidation products such as MDA are formed in terminal phase due to cell damage and are frequently used as biomarkers for overall lipid peroxidation [27]. This process is illustrated by the increase of the MDA and membrane cholesterol concentrations due to the CCl_4_-induced oxidative stress on the RBCs membrane (Table 4). A reverse effect was observed when rats were previously given *Harungana madagascariensis* extracts. According to Hubbell and McConnell [28], the intoxication of experimental animals with carbon tetrachloride altered membrane structures with the increase of MDA and membrane cholesterol concentrations. Furthermore, the altered membrane structures generally lead to the disturbance of their fluidity associated with loss of enzymatic activity and decrease in transport capacity [10,29]. It becomes obvious that pre-treatment of experimental animals with *Harungana madagascariensis* extract could prevent the alteration of membrane fluidity. This was confirmed by the RBCs’ morphology when pre-incubated and incubated with extract and CCl_4_ respectively (Figure 1B and 1F), in addition to the elevated percentage of healthy red blood cells presented in Table 2. Similarly, previous works have shown that silymarin (flavonoid) as well as the extract of *A. deliciosa* reduced the level of H_2_O_2_ - induced stress through the stabilization of MDA concentration [30,31]*.* Thus *Harungana madagascariensis* could protect membrane from lipid peroxidation by inhibiting the free radicals’ attack on bio-membranes. A lower RBCs damage observed on Figure 1D compared to Table 2 could be explained by the antioxidant properties of the Oo. Some abnormalities observed on many RBCs membranes (Figure 1E and 1F) could be referred to the aquaporin blocked by the PCMB [32]. The lesser destruction of RBCs showed in Figure 1F could be attributed to the protective action of the extract.

The function of antioxidant enzymes such as CAT and SOD is to protect cells from toxic reactive oxygen species [33]. In this study, we also determined the enzymatic antioxidant capacity in rat erythrocytes. The pre-administration of the extract stabilizes the activities of CAT and SOD as compared to the control receiving Oo. This result could reinforce the protective effect of the extract on the RBCs membrane. It corroborates with previous works showing that pre-treatment of experimental animals with *Hibiscus cannabinus* extract prevented changes in the CAT and SOD activity as well as the decrease of the membrane cholesterol concentration [34]. It is known that alteration of bio-membranes can affect the membrane permeability [29]. Since an important characteristic of the water permeability of erythrocytes is its inhibition by sulfhydryl - binding mercurial reagents, the RBCs membrane permeability in the presence of PCMB was determined in order to investigate furthermore the extract activity through the membrane [5,35]. At 30°C (Figure 2), the RBCs’ water permeability inhibition percentage induced by PCMB varies from 31.6% (RBCs + extract + PCMB) to 36% (RBCs + PCMB). This could explain a diminished influence of the extract upon the RBC membranes in which the aquaporins are blocked by PCMB at 30°C, which could be due to the *Pd* values of the group RBCs + extract + PCMB which vary from 3.4±1.04 × 10^3^ cm/s (30°C) to 4.9±1.04 × 10^3^ cm/s (37°C) and are closer to the control than those of the group RBCs + PCMB varying from 3.2±1.18 × 10^3^ cm/s (30°C) to 4.2±1.18 × 10^3^ cm/s (37°C).

Furthermore, results showed a remarkable decrease of the inhibition percentage at 25°C and 37°C when RBCs were pre-incubated with the extract, from 33.2% to 25.4% at 25°C and from 31.9% to 19.3% at 37°C respectively. In addition, *Ea* decreased from 30.52 ± 1.3 KJ/mol to 25.49 ± 1.84 KJ /mol. When referring to Figure 1F, where RBCs were less damaged when previously incubated with the extract before adding the PCMB, inhibition percentages obtained could refer to the positive effect of the extract against the aquaporin blockage by PCMB at 25°C and 37°C since it is known that the PCMB molecule blocks the protein channel responsible for the RBC membrane water permeability [32]. These results correlate with data in Table 4 (variation of membrane cholesterol and MDA levels) explaining the protective effect of this extract on the RBCs membrane. According to Schafer and Andreoli, there are two components of the activation energy for water diffusion across the membrane [33]. One is the energy requirement for a water molecule to break the hydrogen bonds formed with neighboring molecules, while the other is the activation energy for water diffusion within the membrane. A low value of *Ea* for a molecule suggests that the molecule’s transport pathway could involve a channel for water diffusion incorporated in membrane proteins with respect to the hydrophobic lipid bilayer. A low value of *Ea* is expected for RBCs with a high permeability and is directly related to the high number of water channels [34]. Subsequently, a slight although non significant increase was observed with the *Pd* (EOo + PCMB) (D) when compared to Pd (PCMB) (F) at 30°C and 37°C. Pd (EOo + PCMB) (D) is closer to control Pd and standard Pd values than Pd (PCMB) (F).

Due to the high specificity techniques deployed results obtained by us were focused on certain parameters and this might be viewed as a limitation of this study. Future work needs to be conducted for further investigation of the mechanism of *Harungana madagascariensis* on the RBCs membrane using complementary techniques. Also the fingerprint of the plant metabolomics needs to be figured out using modern equipment/techniques such as high performance thin layer chromatography (HPTLC).

## Conclusions

According to previous works concerning the antioxidant as well as the antianemic properties of the extract of *Harungana madagascariensis*, it is clear through the present study that this extract could really protect the RBCs membrane and could thus strengthen the priority given to traditional medicine, helping people suffering from different type of anemia such as sickle cells disease.

## Abbreviations

AQP: Aquaporins; BSA: Bovine serum albumin; CAT: Catalase; CCl4: Carbon tetrachloride; Ea: Activation energy; EDTA: Ethylene diamine tetra-acetic acid; HE: Hereditary ellipcytosis; HEPES: (4-(2-hydroxyethyl)-1-piperazineethanesulfonic acid); HNC: National Herbarium of Cameroon; HPP: Hereditary pyropoikilocytosis; HPTLC: High performance thin layer chromatography; HS: Hereditary spherocytosis; MDA: Malondialdehyde; MnCl2: Manganese (II) chloride; NaCl: Sodium chloride; NMR: Nuclear Magnetic Resonance; Oo: Olive oil; PCMB: *Para*- chloromercuribenzoic acid; Pd: Water Diffusional permeability; PHZ: Phenylhydrazine; RBC: Red blood cell; SAO: Southeast Asian Ovalocytosis; SOD: Superoxide dismutase; TBA: Thiobarbituric acid; T2a: ^1^H^+^ longitudinal relaxation time; T2i: ^1^H^+^ transversal relaxation time; Te: Water exchange time; WHO: World Health Organization.

## Competing interests

The authors declare that they have no competing interests.

## Authors’ contributions

BPC carried out the study, the statistical analysis and prepared the manuscript; BS prepared the RBCs samples for the NMR measurements, performed the NMR measurements and calculations and prepared various parts of the manuscript; HM, NYJ and JO co-directed and provided reagents. All the authors read and approved the final manuscript.

## Pre-publication history

The pre-publication history for this paper can be accessed here:

http://www.biomedcentral.com/1472-6882/13/98/prepub
